# Insights into the karyotype and genome evolution of haplogyne spiders indicate a polyploid origin of lineage with holokinetic chromosomes

**DOI:** 10.1038/s41598-019-39034-3

**Published:** 2019-02-28

**Authors:** Jiří Král, Martin Forman, Tereza Kořínková, Azucena C. Reyes Lerma, Charles R. Haddad, Jana Musilová, Milan Řezáč, Ivalú M. Ávila Herrera, Shefali Thakur, Ansie S. Dippenaar-Schoeman, František Marec, Lucie Horová, Petr Bureš

**Affiliations:** 1 Department of Genetics and Microbiology, Faculty of Science, Charles University, Viničná 5, 128 44 Prague 2, Czech Republic; 2 Department of Zoology and Entomology, University of the Free State, P.O. Box 339, Bloemfontein, 9300 South Africa; 3 Crop Research Institute, Drnovská 73, 161 06 Prague 6, Ruzyně Czech Republic; 4 Department of Zoology and Centre for Invasion Biology, University of Venda, Thohoyandou, 0950 South Africa; 5 Biology Centre of the Czech Academy of Sciences, Institute of Entomology, Branišovská 31, 370 05 České Budějovice, Czech Republic; 6 Department of Botany and Zoology, Faculty of Science, Masaryk University, Kotlářská 2, 611 37 Brno Czech Republic

## Abstract

Spiders are an ancient and extremely diverse animal order. They show a considerable diversity of genome sizes, karyotypes and sex chromosomes, which makes them promising models to analyse the evolution of these traits. Our study is focused on the evolution of the genome and chromosomes in haplogyne spiders with holokinetic chromosomes. Although holokinetic chromosomes in spiders were discovered a long time ago, information on their distribution and evolution in these arthropods is very limited. Here we show that holokinetic chromosomes are an autapomorphy of the superfamily Dysderoidea. According to our hypothesis, the karyotype of ancestral Dysderoidea comprised three autosome pairs and a single X chromosome. The subsequent evolution has frequently included inverted meiosis of the sex chromosome and an increase of 2*n*. We demonstrate that caponiids, a sister clade to Dysderoidea, have enormous genomes and high diploid and sex chromosome numbers. This pattern suggests a polyploid event in the ancestors of caponiids. Holokinetic chromosomes could have arisen by subsequent multiple chromosome fusions and a considerable reduction of the genome size. We propose that spider sex chromosomes probably do not pose a major barrier to polyploidy due to specific mechanisms that promote the integration of sex chromosome copies into the genome.

## Introduction

Spiders (Araneae) are a highly diverse animal order, yet the evolution of their genomes and chromosomes is not satisfactorily understood. Most data concern entelegyne araneomorphs, which are the most diversified spider clade. Knowledge of the other main spider lineages (mesotheles, mygalomorphs, haplogyne araneomorphs) is relatively limited (see Supplementary Fig. S1 for spider phylogeny). Available data suggest a considerable diversity of spider genomes and karyotypes. Evolution of spider genomes has also included specific events, such as ancient genome duplication^1^ or formation of peculiar sex chromosome systems, including multiple X chromosomes^2–6^.

The present study is focused on another specific aspect of spider genome evolution, namely karyotype and genome changes associated with the transition from a monocentric (i.e., standard) to a holokinetic (i.e., holocentric) chromosome structure. Although holokinetic chromosomes were discovered more than fifty years ago in some spiders^7^, information on the distribution of these chromosomes across spider phylogeny is very limited.

Holokinetic chromosomes have repeatedly evolved in some protista, plants and invertebrates^8^. Although organisms with holokinetic chromosomes are considered relatively rare, clades possessing such chromosomal structure include more than 350 000 species in total. The bulk of this diversity is formed by four broad animal clades, namely acariform mites, moths and butterflies + caddis flies, hemipteroid insects (Hemiptera and several closely related orders), and some nematode lineages^9^. As for the arachnids, holokinetic chromosomes arose three times, namely within spiders^10^ as well as the ancestors of acariform mites^11^ and buthid scorpions (see the database^12^ for papers dealing with buthid cytogenetics).

Holokinetic chromosomes lack a localised centromere and centromeric connection of the chromatids^13^. Other noteworthy features of holokinetic chromosomes are the low recombination frequency^14^ and regular segregation of most chromosome fragments and fused chromosomes. This unusual pattern of segregation is due to the specific distribution of structures binding microtubules on the chromosome surface during division. Spindle microtubules are attached to the major part of the chromosome poleward surface during mitosis^15^. Holokinetic chromosomes show a considerable diversity of meiotic segregation patterns. Some taxa exhibit the same pattern of microtubule attachment as during mitosis, which enables the segregation of sister chromatids during the first meiotic division (so-called inverted meiosis). Alternatively, microtubules can insert into a telomere region, which is manifested by kinetic activity of this area during segregation (so-called telokinetic meiosis)^15–17^. The occurrence of holokinetic chromosomes in several unrelated groups suggests that they have arisen by a relatively simple, yet unexplained mechanism^9^. The mechanism of monocentric-holokinetic chromosome transition is, however, unresolved.

Spiders with holokinetic chromosomes belong to the haplogyne araneomorphs. This clade contains more than 5 300 species^18^ placed into 18 families^19^. Most haplogynes exhibit monocentric chromosomes^10^. Holokinetic chromosomes have been found in the families Dysderidae and Segestriidae^7^, members of the superfamily Dysderoidea, a species-rich clade comprising nearly 2 700 species^18^. This group also includes the families Oonopidae and Orsolobidae, for which no cytogenetic data are known so far. Palaeontological, biogeographical, and phylogenomic data indicate that Dysderoidea is a relatively ancient group: their fossil records are known from the Cretaceous^20^, orsolobids seem to have spread before the fragmentation of Gondwana (see^21^ for their range), and phylogenomics indicate a Triassic origin of Dysderoidea^22^. In the phylogenomic analyses of spider phylogeny, Dysderoidea is grouped with the families Caponiidae (119 species) and Trogloraptoridae (one species), which together form a sister clade of Dysderoidea^18,19^. Caponiids consist of two clades, Caponiinae and Nopinae. Caponiid and trogloraptorid chromosomes have never been studied.

To reconstruct karyotype evolution in holokinetic spiders, we studied the karyotypes and sex chromosomes of these spiders and their relatives with standard chromosomes. Recent studies indicate that the origin and the evolution of plant holokinetic chromosomes have been accompanied by considerable genome changes (genome size and genome GC proportion), which could be related to the peculiar structure of holokinetic chromosomes^8,23^. Studies on the genome evolution of animals with holokinetic chromosomes are missing. Therefore, we have also analysed the evolution of fundamental genome parameters in haplogyne spiders, including holokinetic groups. Information on these parameters is almost (genome size) or even completely lacking (GC content) in haplogynes. Our results suggest specific traits of karyotype and genome evolution in holokinetic spiders and their close relatives.

## Results

### Karyotypes and sex chromosomes

#### Dysderoidea

**Figure 1:**
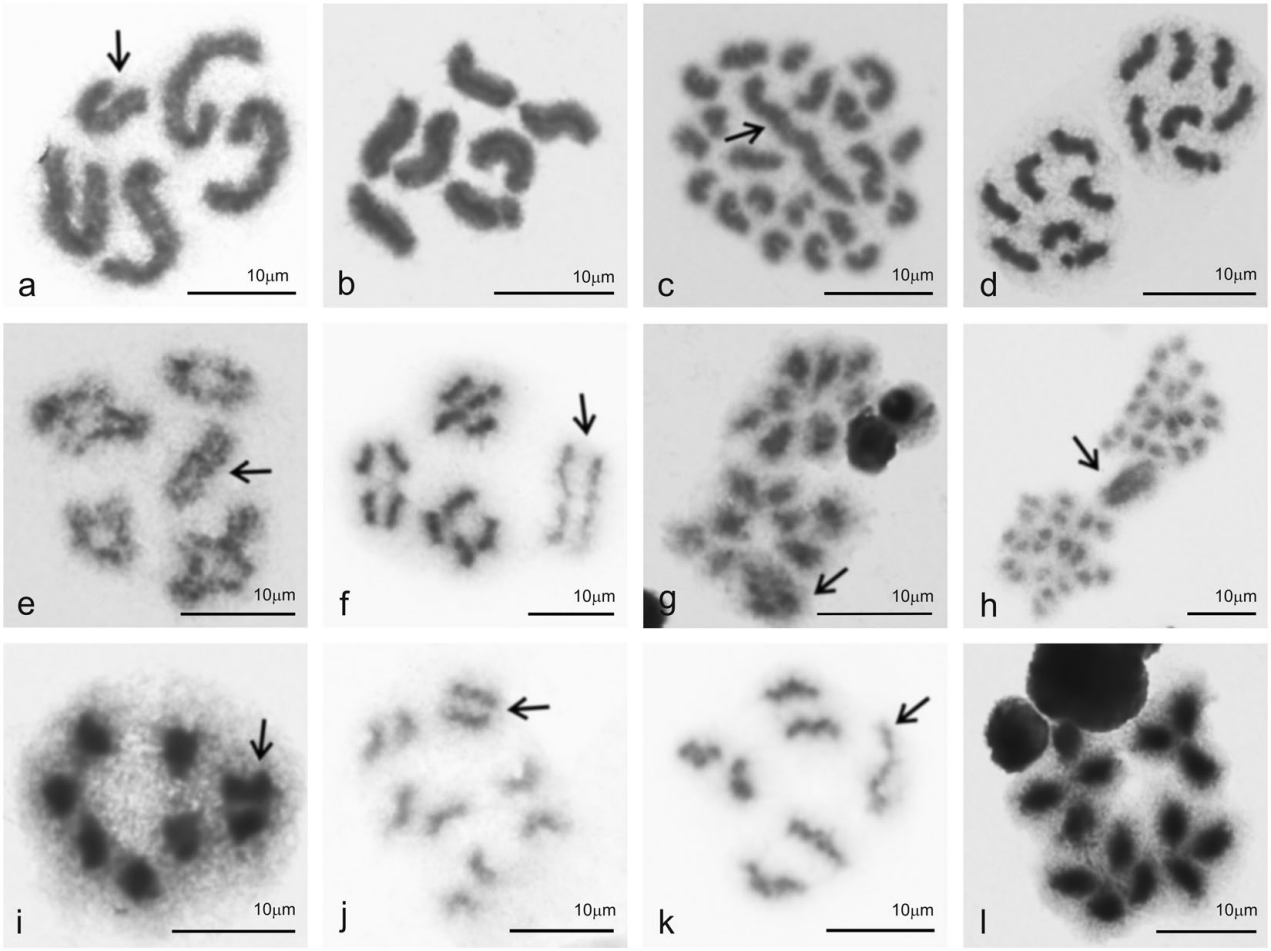
Spiders with holokinetic chromosomes, male mitosis and meiosis. Symbol: **↑** (sex chromosome X). (**a**) *Afrilobus* sp., Orsolobidae (2*n* = 5, X0), mitotic metaphase, the sex chromosome is the smallest element of the karyotype; (**b**) *Gamasomorpha lutzi*, Oonopidae, mitotic metaphase (2*n* = 7, X0), all chromosomes have similar length; (**c**) *Dysderocrates storkani*, Dysderidae, mitotic metaphase (2*n* = 21, X0), note the considerable length of the sex chromosome; (**d**) *G*. *lutzi*, mitotic anaphase; **(e)** *Harpactocrates* sp., Dysderidae, diakinesis plate formed by four bivalents and a sex chromosome (2*n* = 9, X0), each bivalent contains a single chiasma; (**f**) *Harpactea cecconii*, Dysderidae, late metaphase I consisisting of three bivalents and an X chromosome (2*n* = 7, X0): chiasmata are already disintegrated, the sex chromosome is less condensed than bivalents; (**g**) *Harpactocrates* sp., anaphase I, chromosomes show telokinetic activity; (**h**) *D*. *storkani*, anaphase I, the sex chromosome is more condensed than autosomes and exhibits a delayed segregation; (**i**) *Ariadna* sp., Segestriidae, metaphase II (n = 4). In contrast to autosomes, chromatids of the sex chromosome are tightly attached; (**j**) *Dysderocrates* sp., Dysderidae, late metaphase II (n = 5): note the arc-shaped morphology of the autosome chromatids, the sex chromosome is more condensed than autosomes; (**k**) *H*. *cecconii*, metaphase II, the sex chromosome is formed by a single chromatid only (result of inverted meiosis of the sex chromosome); (**l**) *Ariadna* sp., anaphase II: the two left half-plates (n = 4) contain each a sex chromosome, the two right half-plates (n = 3) are without this element. Chromosomes exhibit telokinetic activity.

Cytogenetic analysis involved ten dysderids, four oonopids, one orsolobid, and one segestriid (Table 1). Their karyotypes contained three or four autosome pairs, except for the orsolobid *Afrilobus* sp. (two pairs) (Fig. 1a), and the dysderids *Dysderocrates storkani* (ten pairs) (Fig. 1c) and *Harpactea lepida* (12 pairs) (Fig. S2j). Males exhibited a single sex chromosome (X0 system) (Table 1, Figs 1a–c,e–k and S2a–g,i–l). In species with three and four autosome pairs, the sex chromosomes and autosomes exhibited a more or less similar size, except for *Dysderocrates* sp., where the sex chromosome was substantially larger (Fig. S2l). A prominent X chromosome was also found in all species with a higher number of pairs (Figs 1c,h and S2j). The sex chromosome of *Afrilobus* was considerably shorter than the autosomes (Fig. 1a).

**Table 1:** Karyotype and genome data of studied species with holokinetic chromosomes.

Taxon	Karyotype data			Genome data					
	Specimens	2*n*	Sex chromosomes	Specimens	No. of replicats PI/DAPI	2C (Mbp)	SD (Mbp)	GC (%)	Standard
**Dysderidae (Dysderinae)**									
*Dysdera erythrina*	P^31^	19	X0	6♀	18/15	7644.367	409.639	39.250	VF
*Dysderocrates storkani*	2♂*♂*	21	X0						
*Dysderocrates* sp.	3♂	9	X0	3♀	9/9	3137.160	155.502	36.458	HS
*Harpactocrates* sp.	1 sad ♂	9	X0						
**Dysderidae (Harpacteinae)**									
*Dasumia crassitibialis*	1♂	7	X0	1♀	3/3	5824.284	36.097	37.088	VF
*Harpactea cecconii*	2♂	7	X0						
*H*. *hentschi*	1♂	25	X0	4♀	13/12	9566.995	421.697	39.335	VF
*H*. *hombergi*	4♂	7	X0						
*H*. *lepida*	6♂	25	X0	5♀	16/9	8223.232	443.848	39.106	VF
*H*. *rubicunda*	2♂	7	X0	6♀	18/12	6243.504	445.455	39.369	VF
**Dysderidae (Rhodinae)**									
*Kaemis* sp.	1♂	7	X0						
**Oonopidae**									
*Gamasomorpha lutzi*	3♂	7	X0						
*Ischnothyreus* sp.	1♂	7	X0						
*Oonops ebenecus*	2♂	7	X0						
*O*. *pulcher*	8♂, 2♀	7 (8♀)	X0	12♀	15/12	6920.369	688.575	36.693	VF
**Orsolobidae**									
*Afrilobus* sp.	1♂	5	X0						
*Azanialobus* sp.				1♀	1/0	3581.915			HS
**Segestriidae**									
*Ariadna* sp.	2♂, 1♀	7 (8♀)	X0	5♀	14/8	16890.657	802.325	39.351	VF
*Segestria bavarica*	P^10^	14	X_1_X_2_0	5♀	16/10	6251.373	128.239	38.488	VF
*S*. *senoculata*	P^10^	14	X_1_X_2_0	5♀	15/15	8043.737	254.691	38.940	VF

Unless otherwise specified, diploid numbers concern males. The karyotype data used were either published (P^X^, superscript marks reference number, see list of references) or determined for the first time in our study (see Results). Abbreviations: 2C – DNA content (diploid chromosome complement), DAPI - 4′,6-diamidino-2-phenylindole, HS – *Homo sapiens*, Mbp – mega base pairs, PI – propidium iodide, sad – subadult, SD – standard deviation, VF – *Vicia faba*.

Chromosomes exhibited a specific morphology and segregation behaviour. They lacked a centromeric connection between the chromatids, which was apparent especially during meiosis (Figs 1f,h–k and S2d–f,k). During mitotic segregation, chromosomes were not pulled to the cell pole by any specific region (Fig. 1d). In contrast, during anaphase I they faced the cell pole by their telomeric areas (Fig. 1g,h). During the second meiotic division, both ends of each chromatid were initially pulled to the same pole in dysderids, oonopids and orsolobids (Fig. 1j). In anaphase II, the kinetic activity was restricted to one chromatid end (Fig. 1l). Another meiotic modification was a precocious chiasma disintegration during metaphase I (Figs 1f and S2d).

The sex chromosome displayed a specific behaviour in the male germline. In dysderid *Dasumia crassitibialis*, it showed a precocious separation of chromatids during spermatogonial mitosis (Fig. S2g). Furthermore, the sex chromosome was often more condensed than the other chromosomes and positively heteropycnotic (i.e. stained more intensively than the other elements) during premeiotic interphase and some meiotic phases (Figs 1h,i and S2a,c,d,f,j,k). On the contrary, it was less condensed than the other elements during some meiotic phases in the dysderids *Kaemis* sp., *Harpactea cecconii* and *Harpactocrates* sp. (Figs 1f,k and S2e). In *Dysderocrates*, X chromosome segregation was delayed during anaphase I (Fig. 1h). In the dysderid subfamily Harpacteinae, chromatids of the X chromosome segregated during anaphase I. As a result, the sex chromosome only consisted of one chromatid at metaphase II (Figs 1k and S2k).

#### Caponiidae

**Figure 2:**
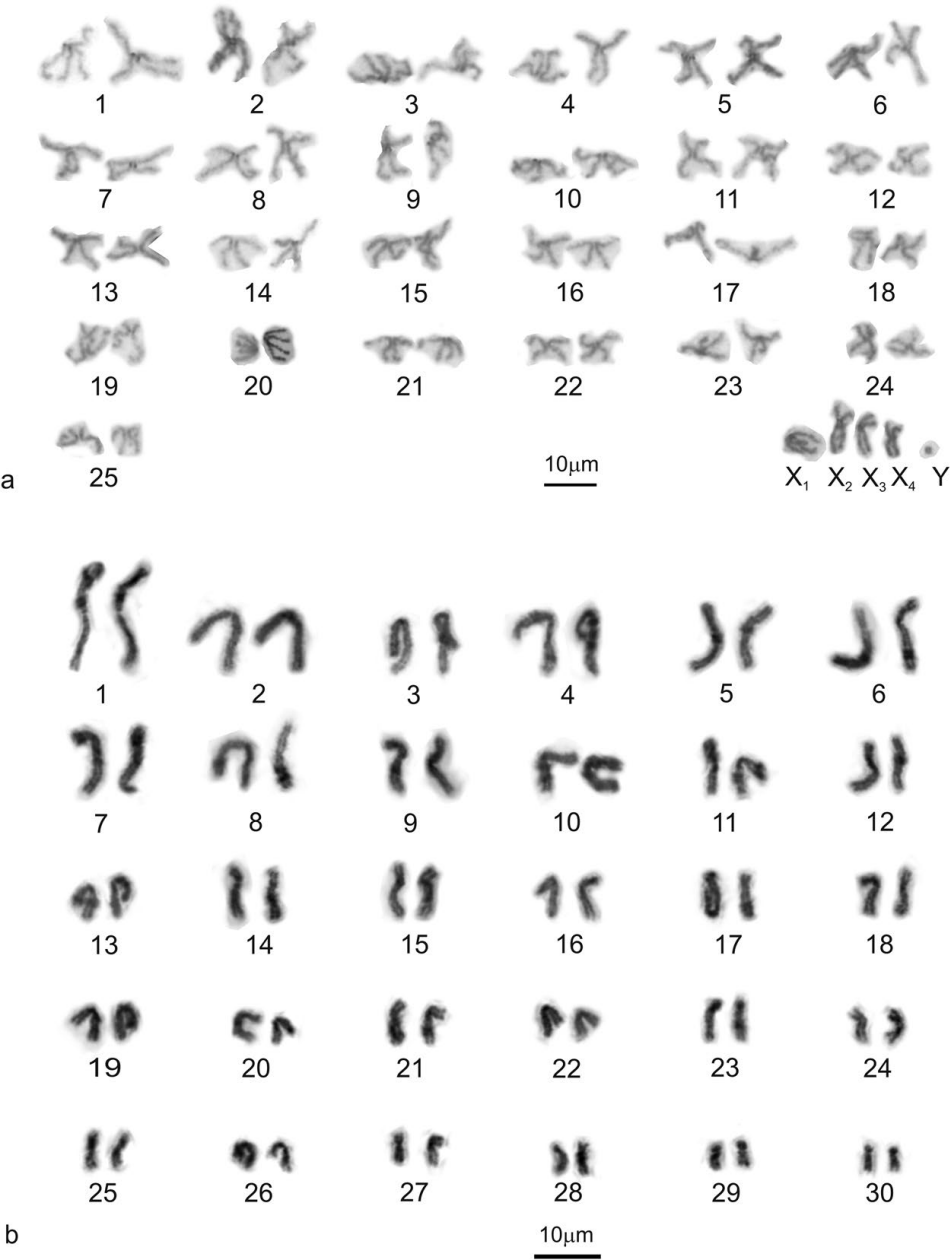
Caponiidae (Nopinae), karyotypes, based on metaphase II **(a)** or mitotic metaphase **(b)**. **(a)** *Nops* aff. *variabilis*, male (2*n* = 55). Chromosome pairs are metacentric except for two submetacentric (nos 18, 25) and subtelocentric pairs (nos 17, 19), sex chromosomes are metacentric except for submetacentric X_2_ and X_3_; **(b)** *Tarsonops* sp., female (2*n* = 60). Chromosome pairs are metacentric except for eight submetacentric (nos 6, 14–16, 18, 26, 28, 29) and five subtelocentric pairs (nos 7, 17, 23, 27, 30).

**Figure 3:**
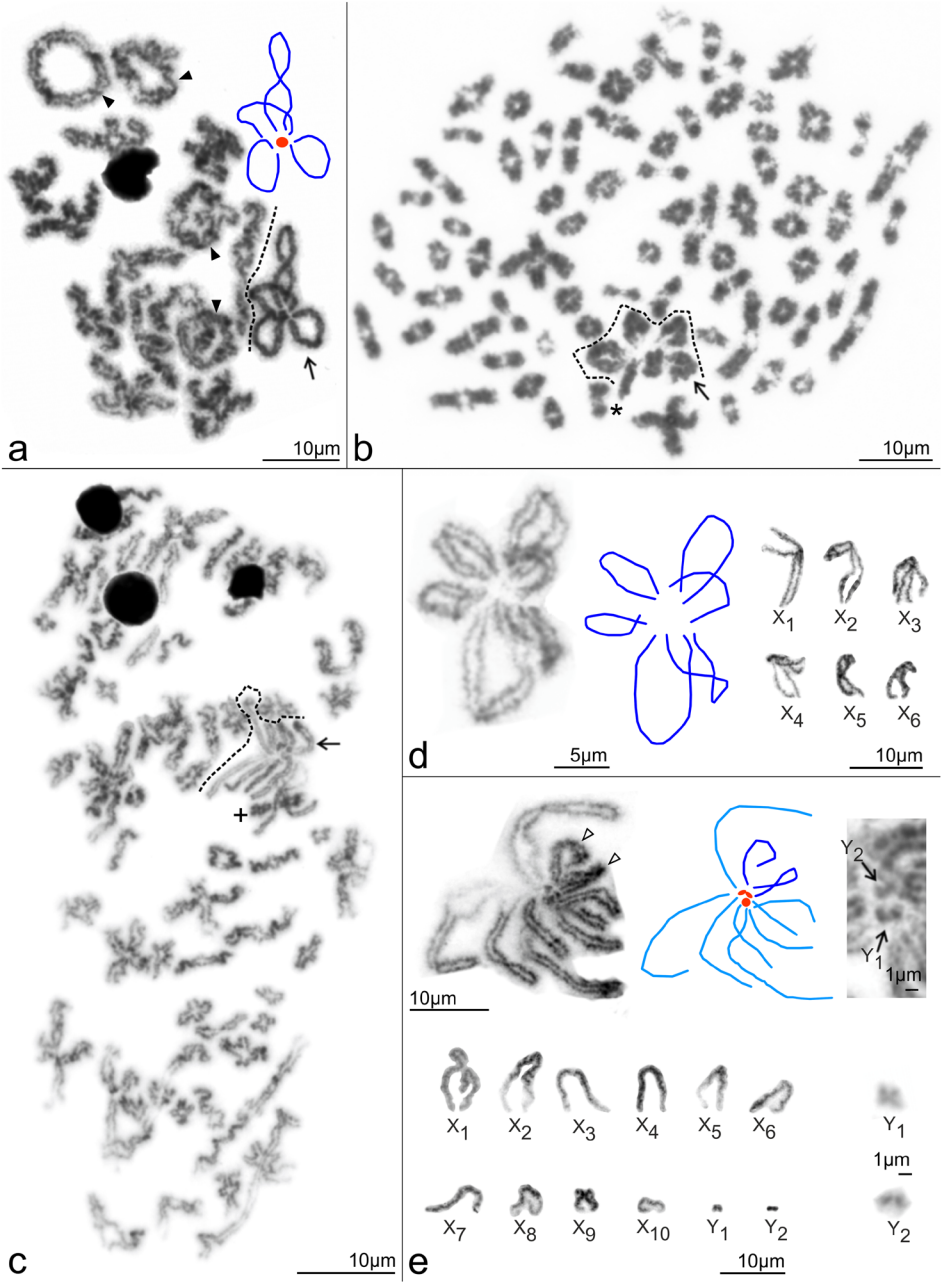
Caponiidae, male meiosis and sex chromosomes. Symbols: **↑** (sex chromosome multivalent), ▲ (bivalent with two chiasmata). Schemes of sex chromosome pairing: dark blue elements – X chromosomes (both chromosome ends involved into pairing); light blue elements – X chromosomes (one chromosome end involved into pairing only); red elements – Y chromosomes. **(a)** *Nops* aff. *variabilis*, incomplete diplotene, X chromosomes are positively heteropycnotic, associated at both ends with a tiny Y chromosome; **(b)** *Caponia natalensis*, metaphase I (73 bivalents and a sex chromosome multivalent, separated from bivalents by dotted line). Each bivalent contains a single chiasma. The sex chromosome cluster is formed by six X chromosomes, which are associated at both ends except for one element (*); **(c)** *C*. *hastifera*, metaphase I (58 bivalents and a sex chromosome multivalent, separated from bivalents by dotted line). Each bivalent contains a single chiasma. The sex chromosome multivalent is intersected by a bivalent (+); **(d)** *C*. *natalensis*, sex chromosomes. From left to right: (1) metaphase I, a sex chromosome cluster composed of six X chromosomes associated at both ends; (2) scheme of sex chromosome pairing; (3) morphology of X chromosomes (metaphase II); **(e)** *C*. *hastifera*, sex chromosomes. First row, from left to right: (1) metaphase I, a sex chromosome cluster formed by 10 X chromosomes and two Y microchromosomes. Both ends of two biarmed X chromosomes (open arrowheads) take part in pairing. In contrast, only one end of the acrocentric X chromosomes is involved in pairing. Tiny Y chromosomes are in the middle of the cluster; (2) scheme of sex chromosome pairing; (3) another metaphase I, centre of multivalent: note the uneven size of the two Y chromosomes. Second row, from left to right: (1) morphology of sex chromosomes (metaphase II); (2) morphology of Y microchromosomes (metaphase II). Note the metacentric Y_1_ chromosome. The Y_2_ chromosome is probably acrocentric.

We obtained data of four representatives of the subfamily Nopinae and three species of the subfamily Caponiinae. Caponiids had substantially higher chromosome numbers than Dysderoidea. The chromosomes of nopines were mostly biarmed, i.e. metacentric and submetacentric (Fig. 2). The male karyotype of *Nops* aff. *variabilis* comprised 55 chromosomes, including four large biarmed X chromosomes and a tiny Y (Fig. 2a); the sex chromosomes were positively heteropycnotic during diplotene (Fig. 3a). The X chromosomes were associated at both ends with the Y chromosome, which was placed in the middle of a sex chromosome cluster. The majority of bivalents contained one chiasma (Fig. 3a). The other species showed a similar 2*n* to *Nops* aff. *variabilis* (Table 2, Figs 2b and S3). *Tarsonops* differed from *Nops* by a higher portion of submetacentric and subtelocentric chromosomes (Fig. 2b).

**Table 2:** Karyotype and genome data of studied species with monocentric chromosomes.

Taxon	Karyotype data			Genome data					
	Specimens	2*n*	Sex chromosomes	Specimens	No. of replicates PI/DAPI	2C (Mbp)	SD (Mbp)	GC (%)	Standard
**Caponiidae (Caponiinae)**									
*Caponia capensis*	1♀	136♀		1♀	3/0	38927.488	1316.477		VF
*C*. *hastifera*	2♂, 1sad♂	128	X_1_-X_10_Y_1_Y_2_	1♀	3/4	47428.569	1475.523	43.183	VF
*C*. *natalensis*	6♂, 1sad♂	152	X_1_-X_6_0						
**Caponiidae (Nopinae)**									
*Nops* aff. *variabilis*	1♂	55	X_1_-X _4_Y	1♂	2/3	31121.376	2956.201	42.246	VF
*Nops* sp.	1♀	62 or 64♀		1♀	3/3	32830.043	2103.421	43.525	VF
*Nopsides ceralbonus*	1♀	64♀							
*Tarsonops* sp.	1♀	60♀							
**Diguetidae**									
*Diguetia albolineata*	P^10^	20	XY	1♀	3/3	2954.346	25.033	43.542	HS
**Filistatidae (Filistatinae)**									
*Filistata insidiatrix*	P^10^	33	X_1_X_2_Y	1♀	3/3	8521.091	174.222	36.342	HS
*Kukulcania* aff. *hibernalis*	P^6^	25	X_1_X_2_Y	1♀	3/3	10259.370	61.477	32.257	HS
*Sahastata nigra*	1♀	28♀		1♀	3/3	11849.197	269.710	33.679	HS
**Filistatidae (Prithinae)**									
*Andoharano ansieae*	3♂	23	X_1_X_2_Y	1♀	3/3	5111.782	333.426	35.899	VF
**Pacullidae**									
*Paculla* sp.	3♂	33	X_1_X_2_Y	4♀	11/12	7658.654	262.096	40.583	VF
**Pholcidae**									
*Pholcus phalangioides*	P^10^	25	X_1_X_2_Y	1♀	3/3	1754.412	48.598	34.555	HS
**Scytodidae**									
*Scytodes* sp. 1	1♂	19	X0	1♀	2/3	4551.007	33.100	42.579	VF
*Scytodes* sp. 2				1♀	3/3	3057.958	149.386	39.826	HS
**Sicariidae**									
*Hexophthalma* sp.	1♀	20♀		1♀	3/3	2587.142	116.078	36.096	HS
*Loxosceles rufescens*	P^10^	21	X_1_X_2_Y	1♀	2/2	10182.792	519.923	39.807	VF

Unless otherwise specified, diploid numbers concern males. The karyotype data used were either published (P^X^, superscript marks reference number, see list of references) or determined for the first time in our study (see Results and Supplementary Figures). Abbreviations: 2C – DNA content (diploid chromosome complement), DAPI - 4′,6-diamidino-2-phenylindole, HS – *Homo sapiens*, Mbp – mega base pairs, PI – propidium iodide, sad – subadult, SD – standard deviation, VF – *Vicia faba*.

The male karyotype of *Caponia natalensis* (2*n*♂ = 152) was composed of 73 autosome pairs and six X chromosomes (Fig. 3b). The sex chromosomes were metacentric, except for submetacentric X_2_ and subtelocentric X_6_ (Fig. 3d). The male karyotype of *C*. *hastifera* (2*n*♂ = 128) consisted of 58 autosome pairs (Fig. 3c), 10 X and 2 uneven tiny Y chromosomes (Fig. 3e). The X chromosomes exhibited acrocentric morphology, except for submetacentric X_1_ and X_9_ (Fig. 3e). The longer Y chromosome was metacentric; morphology of the other Y chromosome was probably acrocentric (Fig. 3e). In *C*. *capensis*, only female mitoses were available (2*n*♀ = 138). The karyotype was slightly predominated by monoarmed (i.e. subtelocentric and acrocentric) chromosomes. Chromosome plates also contained two uneven chromosome fragments (Supplementary Fig. S4). Stability of their number suggests their centric nature.

During male meiosis, *Caponia* bivalents contained a single chiasma (Fig. 3b,c). Sex chromosomes were positively heteropycnotic at some metaphases and anaphases I. The X chromosomes exhibited an end-to-end association. In *C*. *natalensis*, both X chromosome ends were involved in pairing (Fig. 3d). In some plates, however, one chromosome participated in pairing by one end only (Fig. 3b). In *C*. *hastifera*, both ends of two non-acrocentric X chromosomes usually took part in pairing. Acrocentric X’s were associated at one end only. Y chromosomes were in the middle of the cluster (Fig. 3e).

#### Genome size and genome GC content

**Figure 4:**
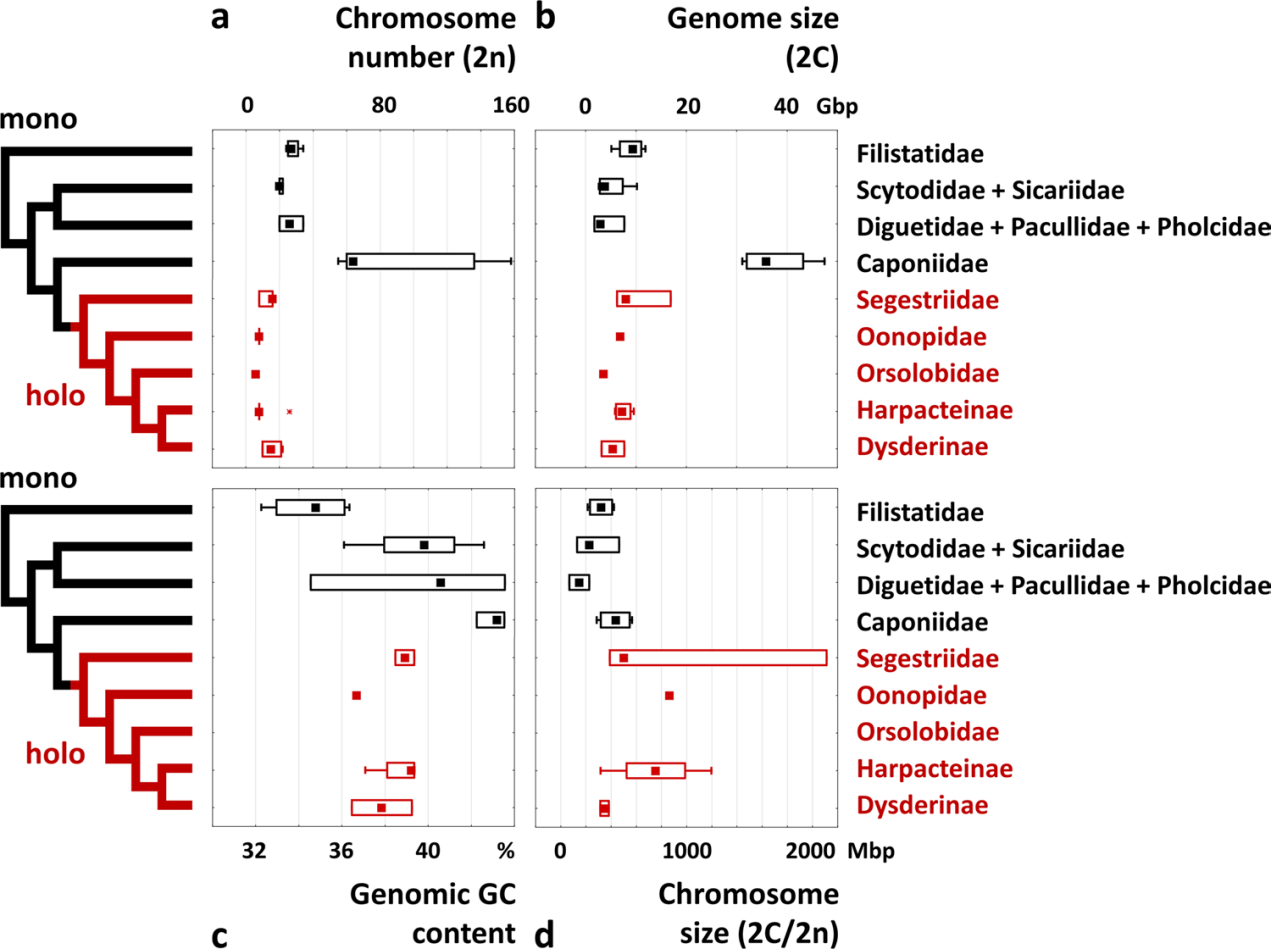
Genome evolution in haplogyne spiders. Karyotype and genome parameters (each species is represented by one value; see Table S3 for the values used) are mapped on the phylogeny of haplogyne spiders (holokinetic clades in red, monocentric in black). The female data are used except *Nops* aff. *variabilis*, in which only male data were available. **(a)** Diploid number of chromosomes (2*n*); **(b)** genome size (2C in Gbp); **(c)** genome GC content (in %); **(d)** average chromosome size (i.e., genome size/chromosome number = 2C/2*n* in Mbp/chromatid). Simplified tree topology is adopted from a phylogenomic study^19^. Boxplots show median (squares), interquartile range (boxes), and non-outlier range (whiskers).

The data on genome size are summarized in Tables 1 and 2 and Fig. 4b. The species of the family Caponiidae had larger genomes (2C = 31.1–47.4 Gbp) than the other haplogyne spiders, which are represented by 2C values varying from 1.8 (*Pholcus*, Pholcidae) to 16.9 Gbp (*Ariadna*, Segestriidae). Among Caponiidae, nopines exhibited smaller genomes (2C = 31.1–32.8 Gbp) than caponiines (38.9–47.4 Gbp). All representatives of the holokinetic clade of Dysderoidea exhibited substantially smaller genomes (2C = 3.1–16.9 Gbp) when compared with their closest relatives, Caponiidae. The average genome size of early-diverging holokinetic spiders (Segestriidae) was considerably higher (10.4 Gbp) than in derived clades of this group (6.4 Gbp). With exclusion of the haplogynes with extraordinarily large genomes (caponiids, *Ariadna*), the average size of holokinetic genomes (6.5 Gbp) was similar to that found in monocentric genomes (6.2 Gbp); the range of sizes of holokinetic genomes was even narrower (3.1–9.6 Gbp) than that of monocentric genomes (1.8–11.8 Gbp). Dysderoidea had larger chromosomes (with average chromatid size 2C/2*n* varying from 314 to 2111 Mbp) when compared with monocentric haplogyne spiders, in which the average chromatid size varied from 67 to 566 Mbp (Fig. 4d).

Genome GC content of haplogynes ranged from 32.3 to 43.5% (Tables 1 and 2, Fig. 4c). This parameter was more variable in lineages with monocentric chromosomes (32.3–43.5%) than in Dysderoidea (36.5–39.4%). Interestingly, some haplogynes exhibited a specific pattern of base ratio. Genomes of a basal haplogyne group, Filistatidae, showed low GC proportion (32.3–36.3%). On the contrary, GC content of caponiids was among the highest found in haplogynes. Moreover, the base ratio of these spiders was very stable (42.2–43.5%). In most haplogynes, species with an increased genome size exhibited a somewhat higher content of CG than their close relatives with a smaller genome (Tables 1 and 2).

## Discussion

**Figure 5:**
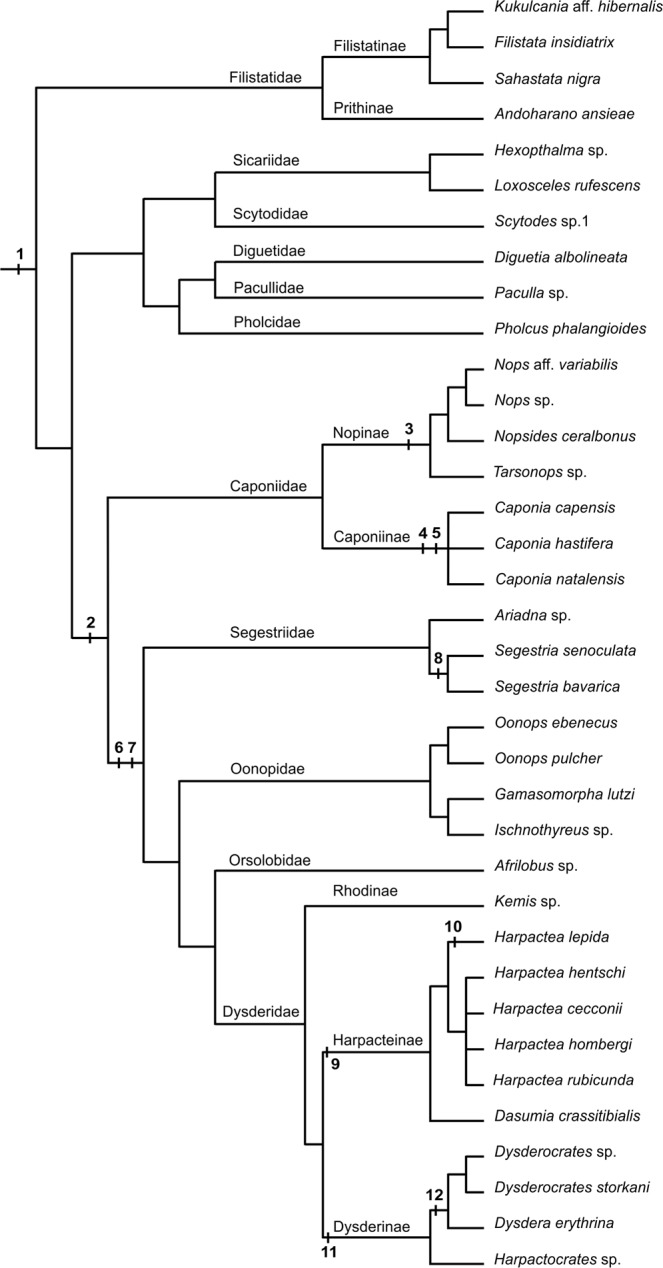
Hypotheses on haplogyne chromosome evolution. Suggested events (numbers in bold): 1 (2*n*♂~40, X_1_X_2_Y; ancestral karyotype of haplogynes), 2 (duplication of genome in common ancestor of Caponiidae and Dysderoidea; the latter includes Segestriidae, Oonopidae, Orsolobidae, and Dysderidae), 3 (X_1_X_2_X_3_X_4_Y_1_Y_2_, ancestral sex chromosome system of Nopinae), 4 (duplication of genome in *Caponia* ancestor), 5 (X_1_X_2_X_3_X_4_X_5_X_6_Y_1_Y_2_, ancestral sex chromosome system of *Caponia*), 6 (origin of holokinetic chromosomes), 7 (2*n*♂ = 7, X0, ancestral karyotype of Dysderoidea), 8 (concerted fission of all chromosomes in ancestor of *Segestria*), 9 (origin of inverted meiosis of sex chromosome in ancestor of Harpacteinae), 10 (2*n*♂ = 25, 2*n* of *Harpactea lepida*), 11 (2*n*♂ = 9, ancestral 2*n* of Dysderinae), 12 (prominent X chromosome, synapomorphy of *Dysdera* and *Dysderocrates*). Tree topology is based on Wheeler *et al*.^19^, except for filistatids (resolved according to Gray^54^), nopines (based on Sánchez-Ruiz & Brescovit^55^), and *Harpactea* (based on cytogenetic data of this study).

Evolution and distribution of holokinetic chromosomes across the phylogeny of spiders are poorly understood. They have so far been proven in the haplogyne families Dysderidae and Segestriidae^7,10,24–27^ belonging to the superfamily Dysderoidea. Our results also suggest a holokinetic chromosome stucture in the other Dysderoidea families, Oonopidae and Orsolobidae. The chromosomes of these families exhibit a specific morphology and segregation, which are typical of holokinetic chromosomes. Caponiids, representing a sister clade to Dysderoidea, have monocentric chromosomes. The holokinetic structure of chromosomes is therefore an autapomorphy of Dysderoidea (Fig. 5).

The available data suggest a considerable diversity of chromosome numbers in Dysderoidea, where 2*n* ranges in males from 5 (*Afrilobus* sp., Orsolobidae)^this study^ to 40 (*Dysdera longirostris*, Dysderidae)^28^. In spite of this, most Dysderoidea exhibit low diploid numbers (2*n*♂ ≤ 20) (see the database^29^, ^this study^). The diploid number of *Afrilobus* is so far the lowest found in spiders. The karyotype of ancestral Dysderoidea featured an extremely low number of chromosome pairs, presumably three pairs, as suggested by the phylogenetic distribution of this pattern. It was found in the basal lineage of Dysderoidea, the family Segestriidae (*Ariadna*)^30^, ^this study^, all primary clades of Dysderidae (Dysderinae, Rhodinae, Harpacteinae)^6,31^, ^this study^, and in various evolutionary lineages of Oonopidae^this study^. Concerning the subfamily Dysderinae, however, three pairs are probably a derived pattern – it was only found in *D*. *septima*^31^. The three chromosome pairs of this species probably arose from a karyotype with four chromosome pairs, a supposedly ancestral feature of the *Dysdera erythrina* group, to which *D*. *septima* belongs^31^. The frequent occurrence of a karyotype with four chromosome pairs in the subfamily Dysderinae^7,27,28,31^, ^this study^ indicates that this pattern might be an ancestral character in this most derived clade within Dysderidae (Fig. 5).

The vast majority of the analysed Dysderoidea feature an X0 sex chromosome system, which is probably ancestral in this group (Fig. 5). The X0 system was also found in many other spider groups, where it arose by chromosome fusion of two or more X chromosomes^5,6^. In *Segestria* (Segestriidae) (2*n*♂ = 14)^29^ and in *Dysdera dolanskyi* (Dysderidae) (2*n*♂ = 20)^31^ the X_1_X_2_0 system was found, which is supposed to be ancestral in spiders^30^. However, it is probably a derived system in spiders with holokinetic chromosomes. We assume that it originated from the X0 system during concerted fissions of all chromosomes in the karyotype (so-called agmatoploidy), which is reflected by the number of chromosome pairs in these spiders being twice as high as in the probable ancestral karyotype of segestriids (2*n*♂ = 7) or the subfamily Dysderinae (2*n*♂ = 9). Such substantial changes in karyotype were discovered in some other organisms with holokinetic chromosomes^13^ but not yet in spiders. In most Dysderoidea with the supposed ancestral karyotype (three autosome pairs, X0 system) (Fig. 5), the length of the X chromosome is approximately the same as the length of autosomes^this study^, which is probably another ancestral feature of this group. A considerable increase in the length of the X chromosome in *Dysderocrates*^this study^ and *Dysdera*^28,31^ is probably their synapomorphy (Fig. 5), and might have been caused by the addition of autosome material.

Holokinetic chromosomes exhibit a specific behaviour during meiosis, related to the different position of the microtubule-binding structures during this division^15–17^. The chromosomes of Dysderoidea, during segregation in the first meiotic division, exhibit telokinetic behaviour that does not occur in their mitosis. The chromosome segregation in the second meiotic division is usually relatively complex. At first, the ends of chromatids begin to move apart, probably as a result of the attachment of microtubules to these regions. Gradually the activity of one chromatid end becomes dominant, so that the chromatid behaviour is again telokinetic. Segregation of the sex chromosome is delayed in the first meiotic division of *Dysderocrates*^this study^ and *Dysdera*^31^. In the sex chromosome univalent of *Dysdera crocata* a peculiar mode of segregation was described, the so-called inverted meiosis^24^, which was also found in some other organisms with holokinetic chromosomes^13^. The sex chromosome of the other *Dysdera* species exhibits standard meiosis^31^. Inverted meiosis is thought to be an adaptation of holokinetic chromosomes to molecular mechanisms of canonical meiosis^32^. The inverted order of meiotic events facilitates proper segregation of chromosome multivalents^33^. The significance of inverted meiosis for segregation of univalents is unresolved. In our study, inverted meiosis of the sex chromosome was found in all representatives of the subfamily Harpacteinae. Therefore, it is probably an apomorphy of this clade (Fig. 5).

Our data revealed considerable diversity of genome sizes in haplogyne spiders, including the holokinetic clade. Although the species diversity of haplogynes is much lower compared to entelegynes, and the data are available for fewer species, the diversity of genome sizes in haplogynes (1.8–47.4 Gbp) is much higher than that found in entelegynes (0.7–5.6 Gbp^34^). In addition, genomes of haplogynes are characterized by considerable diversity of GC content. An increase in genome size in these spiders is often accompanied by an increase in GC content (Fig. 4c), which could reflect the expansion of GC-rich repeats.

A comparison of genome parameters in holokinetic haplogynes and their monocentric relatives allows us to specify genome changes accompanying the origin of holokinetic chromosomes and their subsequent evolution. The genome size and the GC content in caponiids, the closest relatives of the holokinetic spiders, increased substantially (Fig. 4b,c). Thus, the origin of holokinetic chromosomes seems to be associated with the genome downsizing and reduction of GC content, i.e. with the same genome changes found in some plant holokinetic clades^8,23^. Some members of the early-diverging holokinetic family Segestriidae have very large genomes compared to derived clades of holokinetic spiders. This pattern suggests that genome reduction continued after the formation of holokinetic chromosome structure. Results in holokinetic plants and spiders indicate that the reduction of genome size and GC content could be an essential component of the evolutionary transition from monocentric to holokinetic chromosomes across eukaryotes. The reduction of GC content could be related to the lower frequency of crossing-over and gene conversion in holokinetic chromosomes^23^; the latter process is GC-biased^35^. Another pattern found in holokinetic haplogynes consistent with the other holokinetic organisms is the increased variation in the chromosome size (Fig. 4d), which could be generated by holokinetic drive during asymmetric female meiosis, when the larger homologues are preferentially transmitted to ovules in some lineages but driven to pole bodies in the other lineages of the same clade^36^. However, such a homolog size-preferring holokinetic drive also results in an inverted relationship between the chromosome number and the genome size in plant holokinetic lineages^36^. No such relationship was found in the holokinetic spiders. In this context, it should be noted that holokinetic drive is not acting in organisms with a telokinetic behaviour of chromosomes during meiosis^36^, a behaviour that is characteristic of male chromosomes of holokinetic spiders. If female chromosomes of these spiders also exhibit telokinetic meiotic behaviour, the increased size variation in the chromosomes of holokinetic spiders would be a consequence of process(es) other than holokinetic drive. With exclusion of extraordinarily large genomes (caponiids, *Ariadna*), the diversity of genome sizes is lower in holokinetic haplogynes than in monocentric haplogynes (Fig. 4b). Similarly, the proportion of GC base pairs is more stable in the evolution of holokinetic spiders than in their close monocentric relatives (Fig. 4c). These unusual patterns may be related to the specific structure of the holokinetic chromosomes and should be tested in other groups with these chromosomes.

Remarkably, caponiids have much larger genomes than other spiders. Concerning other arthropods, genomes exceeding caponiid genomes in size have only been found in some crustaceans^37^. The extreme genome sizes in caponiids could result from polyploidization. Although genome duplications were less frequent in animal evolution than in other organisms, they have occurred in spiders, as shown recently. Phylogenomic analysis revealed a polyploid event in spider ancestors^1^. The specific constitution of sex chromosomes in the mygalomorph superfamily Avicularioidea indicates an additional polyploid event in spiders, namely in the ancestors of these mygalomorphs^38^. Therefore, we also explored the karyotypes of caponiids to find specific features that would support the hypothesis of polyploid origin of these spiders.

Diploid numbers of caponiids are considerably higher than in other haplogynes with monocentric chromosomes, which have male 2*n* from 9 (*Micropholcus* spp., Pholcidae^39^) to 37 (*Izithunzi capensis*, Drymusidae^10^). Based on diploid numbers, caponiids can be divided into two groups. Nopines exhibit lower and conservative chromosome numbers (Table 2), while caponiines have chromosome numbers at least twice as high as nopines. *Caponia natalensis* (2*n*♂ = 152) has the highest chromosome number so far known among spiders.

Nopines, like other monocentric haplogynes, exhibit predomination of biarmed chromosomes, which is probably a symplesiomorphy of araneomorph and mygalomorph spiders^38^. Although *Nops* and *Tarsonops* comprise a similar 2*n*, they differ considerably in the proportion of monoarmed chromosomes, which indicates differentiation of nopine karyotypes by rearrangements changing chromosome morphology (i.e. pericentric inversions, some variants of translocations). *C*. *capensis* exhibits a slight predomination of monoarmed chromosomes, which could have arisen from ancestral biarmed chromosomes through pericentric inversions or centric fissons. The latter scenario is supported by centric fragments in the karyotype of this species, which could arise during fissions of monocentric chromosomes^40^.

**Figure 6:**
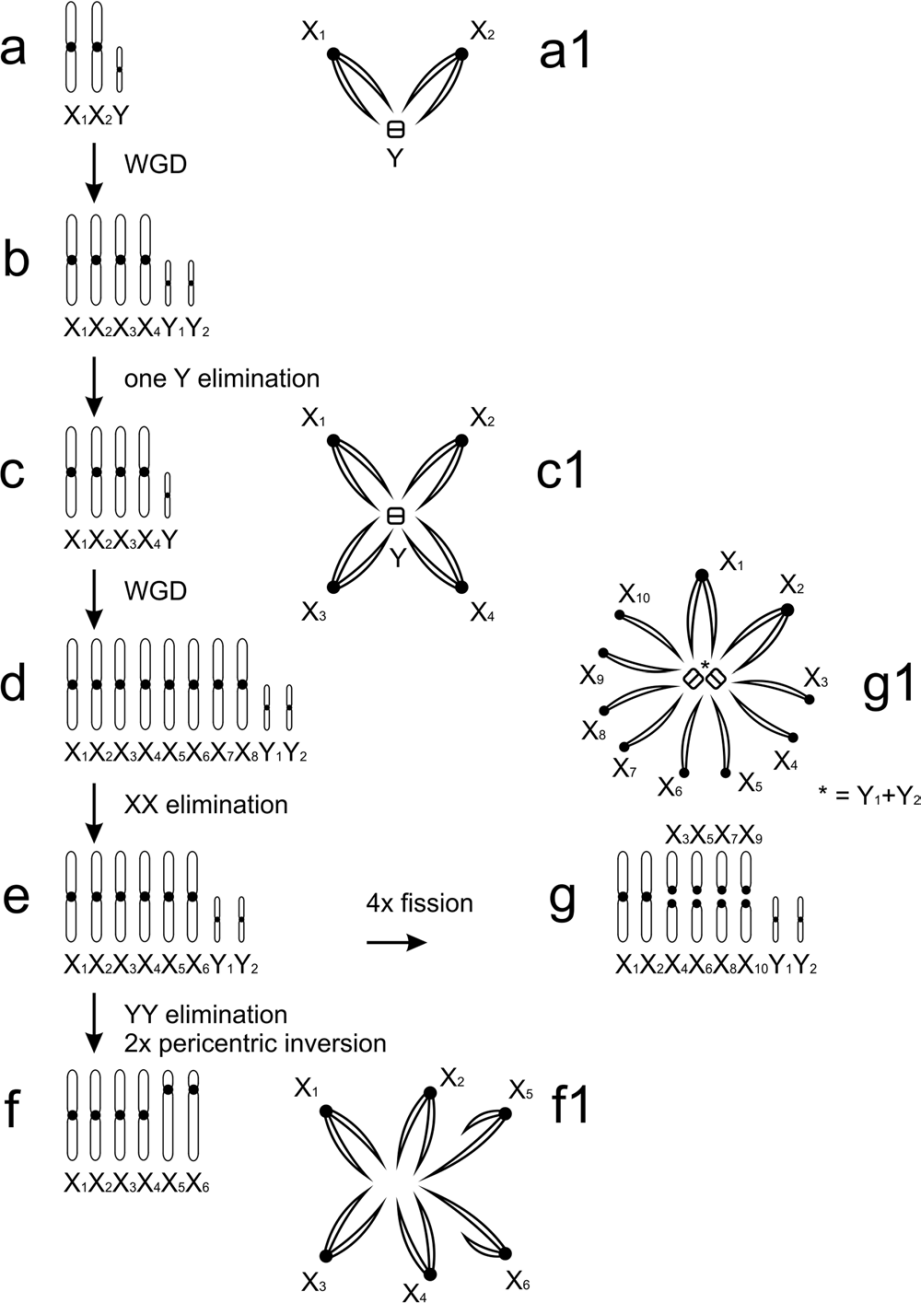
Caponiidae, hypothesis of sex chromosome evolution. Abbreviation: WGD (whole genome duplication). **(a)** diploid ancestor of caponiids (a_1_: sex chromosome pairing, male meiosis); **(b)** ancestor of supposed tetraploid lineage; **(c)** *Nops* aff. variabilis (c_1_: sex chromosome pairing, male meiosis); **(d)** ancestor of *Caponia* lineage; **(e)** ancestral karyotype of *Caponia*; **(f)** *C*. *natalensis* (f_1_: sex chromosome pairing, male meiosis); **(g)** *C*. *hastifera* (g_1_: sex chromosome pairing, male meiosis).

Caponiid sex chromosome systems are complex and involve much higher numbers of chromosomes than those of other haplogynes. Despite this, the caponiid sex chromosomes retain a peculiar achiasmatic pairing during male meiosis, which is common in other haplogynes. The sex chromosome system of *Nops* (X_1_X_2_X_3_X_4_Y) can be inferred from the X_1_X_2_Y system, which has been found in a number of haplogyne families^10,41,42^^, this study^, and which is probably ancestral in araneomorph spiders^42^, including haplogynes (Fig. 5). The ancestral X_1_X_2_Y system probably consisted of two large metacentric X chromosomes and a microchromosome Y^10^ (Fig. 6a). In male meiosis, the X chromosomes pair achiasmatically by their ends with the Y chromosome^10^ (Figs 6a1 and S5b,d). The X_1_X_2_X_3_X_4_Y system of *Nops* could have arisen by a duplication of the X_1_X_2_Y system (Fig. 6b), as the morphology and meiotic pairing of the chromosomes are the same, followed by elimination of one Y chromosome (Fig. 6c). The sex chromosome system of *C*. *natalensis* (six biarmed X chromosomes, which again associate by their ends during male meiosis, Fig. 6f,f1) arose from the ancestral sex chromosome constitution of *Caponia* (Fig. 6e) by the loss of two Y microchromosomes. The sex chromosomes of *C*. *hastifera* form one of the most complex sex chromosome systems found so far: it is composed of ten mostly acrocentric X chromosomes and two Y microchromosomes (X_1_X_2_X_3_X_4_X_5_X_6_X_7_X_8_X_9_X_10_Y_1_Y_2_) (Fig. 6g). Acrocentric Xs probably arose from the ancestral biarmed sex chromosomes of *Caponia* (Fig. 6e) by centric fissions (Fig. 6g). Acrocentric Xs participate in pairing by one end only (Fig. 6g1), which was also observed in achiasmatic monoarmed Xs of other haplogynes^10^.

The considerable increase of DNA content and 2*n*, and the possible duplication of sex chromosomes, support genome polyploidisation in caponiid ancestors. The relatively high frequency of polyploid events in the evolution of spiders is surprising, given that their genomes comprise complex sex chromosome systems. In general, sex chromosomes constitute a major barrier to maintaining polyploid genomes. Their duplications lead to a disruption of sex-determining mechanisms and dosage compensation^43^, especially in organisms with a high degree of sex chromosome differentiation, such as spiders. Available data, however, suggest the integration of sex chromosome copies arising by nondisjunctions into the spider genome^4,44,45^, which predict specific mechanisms to cope with dosage disruption caused by extra chromosome copies. Maintaining the sex chromosome copies could be facilitated by the unique behaviour of spider sex chromosomes during meiosis of the heterogametic^5,6,38^ and homogametic sex^4,45^, which probably hampers the pairing of structurally similar (i.e. homeologous) chromosomes and facilitates the structural differentiation of newly formed sex chromosomes. Mechanisms promoting the integration of the sex chromosome copies into the genome of spiders could facilitate the establishment of polyploidy in these animals.

Interestingly, the smaller sizes of nopine genomes correlate with lower 2*n* and lower numbers of Xs and Ys. One possible explanation could be that nopines have undergone one, while *Caponia* two, genome duplications (in such a case the *Caponia* genome would be octoploid) (Fig. 5). The duplication of sex chromosomes in *Nops* would lead to the rise of eight biarmed X chromosomes and two Y chromosomes (Fig. 6d). However, our results suggest that the ancestral *Caponia* karyotype included only six biarmed X chromosomes (Fig. 6e). The lower number of X chromosomes might be caused by the loss of chromosomes, which often occurs after the induction of polyploidy^46^. Such events are probable, especially in sex chromosomes. Despite the possible tolerance of the spider genomes to the presence of sex chromosome copies, their high number might be detrimental.

The polyploid event might have already occurred in the common ancestor of caponiids and holokinetic spiders. If so, the origin of spider holokinetic chromosomes can be understood as a specific mode of differentiation of the duplicated genome. Polyploid events are followed by a genome reduction, which often includes multiple chromosome fusions and the loss of a considerable amount of DNA, including the coding sequences. Multiple chromosome fusions could promote the spreading of microtubule-binding structures over a major part of the chromosomes, which is a specific feature of holokinetic chromosomes. The differentiation of a polyploid genome could even be a relatively common process for the origin of holokinetic chromosomes. For example, this could also be a possible explanation for the origin of holokinetic chromosomes in scorpions. The genome of the scorpion ancestors also underwent a duplication^1^. As in spiders, the genomes of holokinetic scorpions also feature much lower 2*n* than their monocentric relatives. Further investigations of caponiids and holokinetic spiders by genomic approaches would allow testing hypotheses about the polyploid events in their evolution, and the possible role of polyploidization in the origin of holokinetic chromosomes.

## Methods

### Chromosome preparations and their evaluation

Most cytogenetic data were obtained from adult males, either from the whole content of the abdomen (oonopids) or only the testes (other haplogynes). The spider testes are usually formed by a pair of tubes. In caponiids and Dysderoidea (except for *Ariadna*), the distal ends of these tubes were fused. Beside spermatogonial mitoses, testes of adult males also contained meiotic cells. Analysis of meiotic plates allowed us to determine the sex chromosomes. In caponiids and *Harpactocrates*, subadult males were available. In subadult caponiids, testes contained only mitoses and prophase I spermatocytes. Female chromosomes were obtained from the ovaries, intestine or abdominal content. In *Caponia*, the proximal parts of the ovaries were fused into a single tube. The morphology of the ovaries in the other caponiids was not determined in this study. Female tissues only contained mitotic plates. Data on the collection and specimens used are presented in Tables 1 and 2 and Supplementary Tables S1 and S3. Dissected specimens are deposited in the collections of J.K., M.R., and A.S.D.S.

Preparation of chromosome slides was based on the protocol of Dolejš *et al*.^47^, except for the prolonged treatment of caponiid tissues (45–50 min) by hypotonic solution (0.075 M KCl), which reflects their considerable resistance to hypotonization. Unless otherwise specified, preparations were stained by Giemsa. Slides were inspected under an Olympus BX 50 microscope. Images were captured using an Olympus DP 71 CCD camera using an oil immersion lens (100x). Chromosome measurements were carried out using the IMAGE TOOL 3.0 programme. Karyotypes were constructed using the Corel PHOTO-PAINT X9 software. Chromosome morphology was based on centromeric index, which was calculated as the ratio of the longer and shorter chromosome arm. Relative chromosome length was estimated as a percentage of the total chromosome length of the diploid set.Genome size measurement and genome GC content estimation

In selected species, genome size was determined by flow cytometry (FCM). Female individuals were preferably measured to prevent fluctuations caused by the different numbers of sex chromosomes. Fresh or frozen specimens (stored at −80 °C without any preservative) were explored. Freezing of arthropod samples does not affect FCM measurements^48^. Legs, or the prosoma with legs (in small species), were selected as the optimal source of nuclei. Due to the high debris content, abdominal tissues were not suitable for these experiments. To prepare samples for FCM, a two-step method was performed^49^. Briefly, sample tissue was chopped together with plant DNA standard (*Vicia faba* ‘cultivar Inovec’; 2C = 23 272.88 Mbp^50^) using a razor blade in cold Otto I buffer (1–3 ml). Alternatively, human male leucocytes (2C = 6055.03 Mbp – the value following human/*Vicia faba* ‘Inovec’ ratio estimated by Doležel *et al*.^51^) were added as DNA standard. The suspension of sample and standard nuclei was subsequently filtered through a 0.2 µm nylon sifter. Finally, Otto II buffer (1–1,5 ml) containing fluorochrome propidium iodide (PI) was mixed with the filtered suspension to stain the nuclei. After incubation of the mixture (at least 20 min, RT, darkness), FCM was performed using cytometers of Partec GmbH (recently Sysmex), CyFlow ML (equipped with 100 mW laser Cobold Samba) or CyFlow SL (200 mW laser Cobold Samba). Each measurement involved 5000 particles. The results were calculated from the resulting histograms showing the relative fluorescence of the sample and standard by FlowMax software (Partec). The average coefficient of variation of all measurements was 6.64%.

Beside the genome size, genome GC content was estimated in most species using FCM, when the previously described estimation of genome size using intercalating fluorochrome propidum iodide was combined with AT selective fluorochrome 4′,6-diamidino-2-phenylindole (DAPI) in parallel analyses of the same samples. Fresh leaves of *V*. *faba* (cultivar Inovec) (GC = 41.15%^50^) or male human leucocytes (GC = 43.60% – the value following human/*Vicia faba* ‘Inovec’ DAPI and PI ratios estimated by Doležel *et al*.^51^) were used as standards. Sample preparation and measurements were the same as described above. Measurements with DAPI were performed using Partec cytometers: PA I (equipped with Mercury HBO lamp) or CyFlow ML (UV-LED). The average coefficient of variation was 2.26%. To calculate the genome GC content, the formula of Barrow and Meistner^52^ was applied using automatic spreadsheet http://www.sci.muni.cz/botany/systemgr/download/Festuca/ATGCFlow.xls^53^.

If possible, more replicates were performed (on different days) using tissues of the same or more individuals. Final values of genome parameters were determined as the average of values of the particular replicates.

## Supplementary information

Dataset 1

## Data Availability

Data generated or analysed during this study are included in this published article (and its Supplementary Information files). Dissected specimens are deposited in the collections of J.K., M.R., and A.S.D.S.
