# Supplementary material for: Insights into the karyotype and genome evolution of haplogyne spiders indicate a polyploid origin of lineage with holokinetic chromosomes
Source: Sci Rep. 2019 Feb 28;9:3001. doi: 10.1038/s41598-019-39034-3 (PMC6395618; doi:10.1038/s41598-019-39034-3)
Supplement: Supplementary file 1 — Dataset 1 [file 41598_2019_39034_MOESM1_ESM.pdf]

Supplementary Figures and Tables

Insights into the karyotype and genome evolution of haplogyne spiders indicate a polyploid origin of lineage with holokinetic chromosomes

Jiří Král[1*], Martin Forman[1], Tereza Kořínková[1], Azucena C. Reyes Lerma[1], Charles R. Haddad[2], Jana Musilová[1,3], Milan Řezáč[3], Ivalú M. Ávila Herrera[1], Shefali Thakur[1], Ansie S. Dippenaar-Schoeman[4], František Marec[5], Lucie Horová[6], Petr Bureš[6]
[1]Department of Genetics and Microbiology, Faculty of Science, Charles University, Viničná 5, 128 44 Prague 2, Czech Republic
[2]Department of Zoology and Entomology, University of the Free State, P.O. Box 339, Bloemfontein 9300, South Africa
[3]Crop Research Institute, Drnovská 73, 161 06 Prague 6 - Ruzyně, Czech Republic
[4]Department of Zoology and Centre for Invasion Biology, University of Venda, Thohoyandou, 0950, South Africa
[5]Biology Centre of the Czech Academy of Sciences, Institute of Entomology, Branišovská 31, 370 05 České Budějovice, Czech Republic
[6]Department of Botany and Zoology, Faculty of Science, Masaryk University, Kotlářská 2, 611 37 Brno, Czech Republic

Supplementary Figure 1. Phylogeny of spiders and related arachnid orders. A compilation of hypotheses from two published studies[19,56].

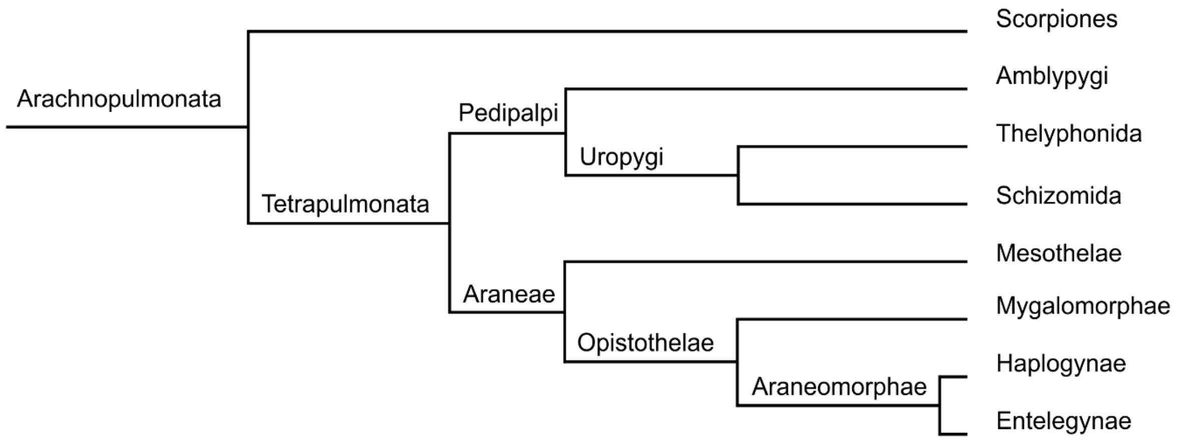

Supplementary Figure 2. Mitosis and meiosis in holokinetic haplogyne spiders of the families Oonopidae and Dysderidae. Preparations were stained with Giemsa except **b** and **c** stained with DAPI. Unless otherwise specified, based on male preparations. Abbreviations and symbols: * (bivalent with two chiasmata), N (interkinesis nucleus), X (X chromosome). **(a)** *Oonops ebenecus*, Oonopidae (2*n* = 7, X0), diplotene consisting of three bivalents and an X chromosome, which is positively heteropycnotic; **(b, c)** *O. pulcher*. (**b**) Mitotic metaphase (2*n* = 7, X0), X chromosome is slightly longer than the other chromosomes; **(c)** Diplotene, note three bivalents and the positively heteropycnotic X chromosome; (**d**) *Ischnothyreus* sp., Oonopidae, late metaphase I (2*n* = 7, X0) after disintegration of chiasmata, consisting of three bivalents and a positively heteropycnotic X chromosome; (**e, f**) *Kaemis* sp., Dysderidae (Rhodinae). (**e**) Diakinesis (2*n* = 7, X0) consisting of three bivalents and an X chromosome exhibiting a low condensation; (**f**) two sister metaphases II, n = 4 (left) and n = 3 (right), separated by a dashed line. The X chromosome differs from the other chromosomes by tighter attachment of the chromatids and slightly positive heteropycnosis; (**g**) *Dasumia crassitibialis*, Dysderidae (Harpacteinae), spermatogonial metaphase (2*n* = 7, X0). The X chromosome exhibits a slightly precocious separation of the chromatids; (**h**) *Harpactea hentschi*, Dysderidae (Harpacteinae), female, oogonial metaphase (2*n* = 8); (**i**) *H. hombergi*, metaphase I (2*n* = 7, X0). Note three bivalents and an X chromosome univalent; (**j**) *H. lepida*, metaphase I (2*n* = 25, X0) consisting of 12 bivalents and a large X chromosome, which is positively heteropycnotic; (**k**) *H. rubicunda*, metaphase II plate (n = 4) and adjacent interkinesis nucleus containing a positively heteropycnotic X chromosome on the periphery. The X chromosome of the metaphase II plate is formed by a single chromatid only as a result of inverted meiosis of the sex chromosome; (**l**) *Dysderocrates* sp.n., Dysderidae (Dysderinae), spermatogonial metaphase (2*n* = 9, X0); the sex chromosome is considerably longer than the other chromosomes.

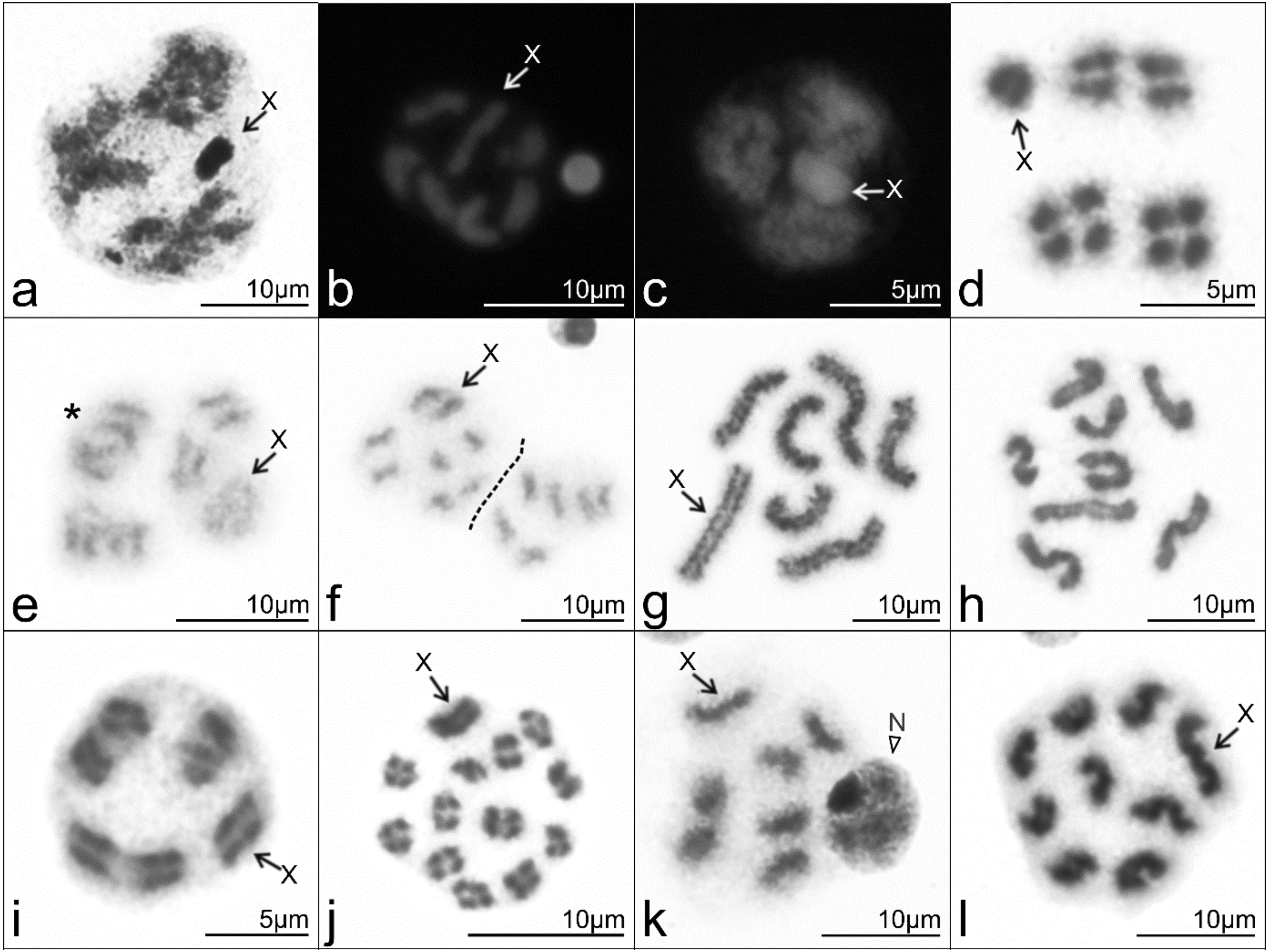
X
a
10μm
X
b
10μm
X
c
5μm
X
d
5μm
*
X
e
10μm
X
f
10μm
X
g
10μm
h
10μm
X
i
5μm
X
j
10μm
X
N
k
10μm
X
l
10μm

Supplementary Figure 3. Mitotic chromosomes of Caponiidae (Nopinae), females. Red dotted lines indicate chromosome contacts, green dotted lines overlapping of chromosomes. (**a**) *Nopsides ceralbonus*, oogonial mitosis (2n = 64). Below the same mitosis with indicated chromosome contacts and overlaps; (**b**) *Nops* sp., oogonial mitosis (2n = 60-62). Below the same mitosis with indicated chromosome contacts and overlaps.

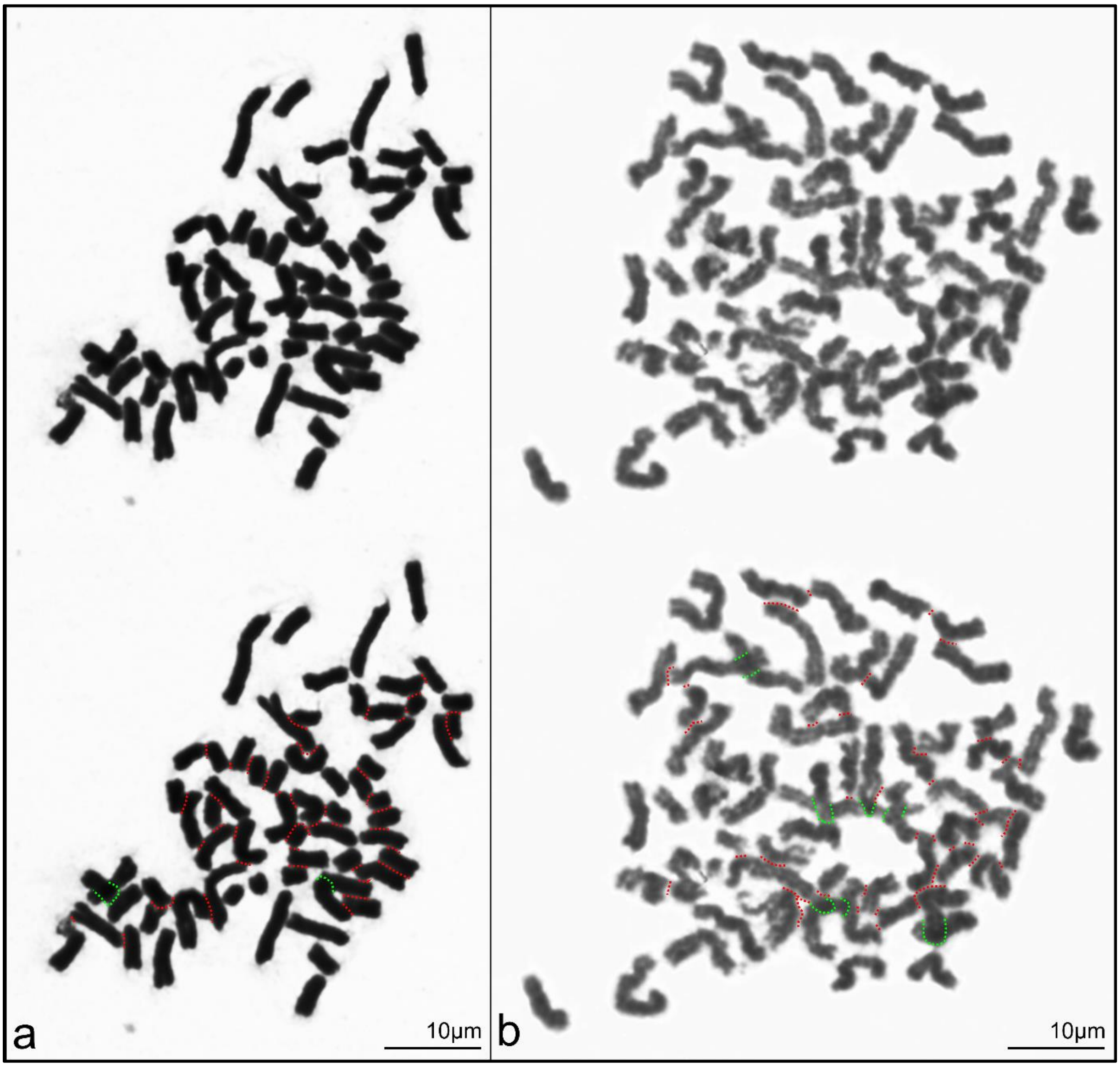

Supplementary Figure 4. Female karyotype of *Caponia capensis* (Caponiidae), based on mitotic metaphase (2*n* = 136). The karyotype consists of 14 metacentric (nos 1-3, 5, 8-10, 13, 24, 45, 53, 55, 63, 68), 12 submetacentric (nos 4, 7, 12, 17, 18, 20, 21, 23, 28, 31, 46, 60), 16 subtelocentric (nos. 6, 11, 14-16, 19, 22, 25, 27, 30, 35-37, 41, 47, 51), and 26 acrocentric pairs (nos. 26, 29, 32-34, 38-40, 42-44, 48-50, 52, 54, 56-59, 61, 62, 64-67). The karyotype of this specimen also contains two tiny uneven chromosome fragments (F).

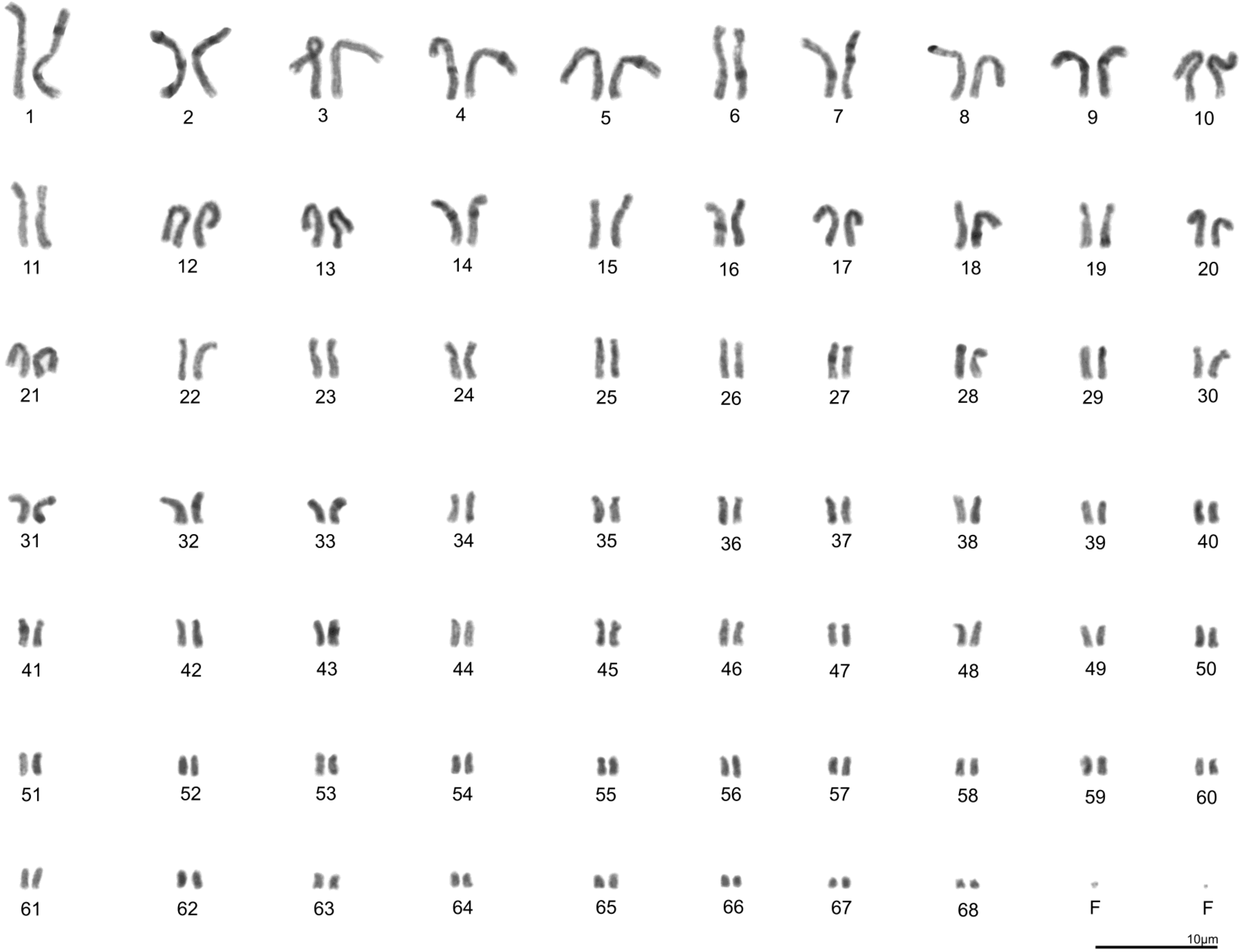
1 2 3 4 5 6 7 8 9 10
11 12 13 14 15 16 17 18 19 20
21 22 23 24 25 26 27 28 29 30
31 32 33 34 35 36 37 38 39 40
41 42 43 44 45 46 47 48 49 50
51 52 53 54 55 56 57 58 59 60
61 62 63 64 65 66 67 68 F F
10μm

Supplementary Figure 5. Chromosomes of monocentric haplogyne spiders used for the analysis of genome size. Unless otherwise specified, based on male preparations. Abbreviations: P (precocious separation of homologous chromosomes of a bivalent), S (sex chromosome trivalent), X (sex chromosome X), Y (sex chromosome Y), S (sex chromosome trivalent). (**a**) *Sahastata nigra*, Filistatidae, female, oogonial metaphase (2n = 28); (**b**) *Andorahano ansieae*, Filistatidae (2n = 25, X_1X_2Y), spermatogonial metaphase containing a tiny Y chromosome. Inset: sex chromosome trivalent in metaphase I. Two large X chromosomes pair achiasmatically by their ends with a Y microchromosome (see schematic drawing); (**c**, **d**) *Paculla* sp., Pacullidae (2n = 33, X_1X_2Y). (**c**) Spermatogonial metaphase without any microchromosome, which implies a relatively large size of the Y chromosome; (**d**) Metaphase I comprising 15 bivalents and a sex chromosome trivalent formed by two X chromosomes and a Y chromosome. In contrast to the previous species, the sex chromosome trivalent is highly condensed. Schematic drawing shows supposed pairing of sex chromosomes in the trivalent; (**e**) *Scytodes* sp. 1, spermatogonial metaphase (2n = 19, X0) containing a large metacentric X chromosome.

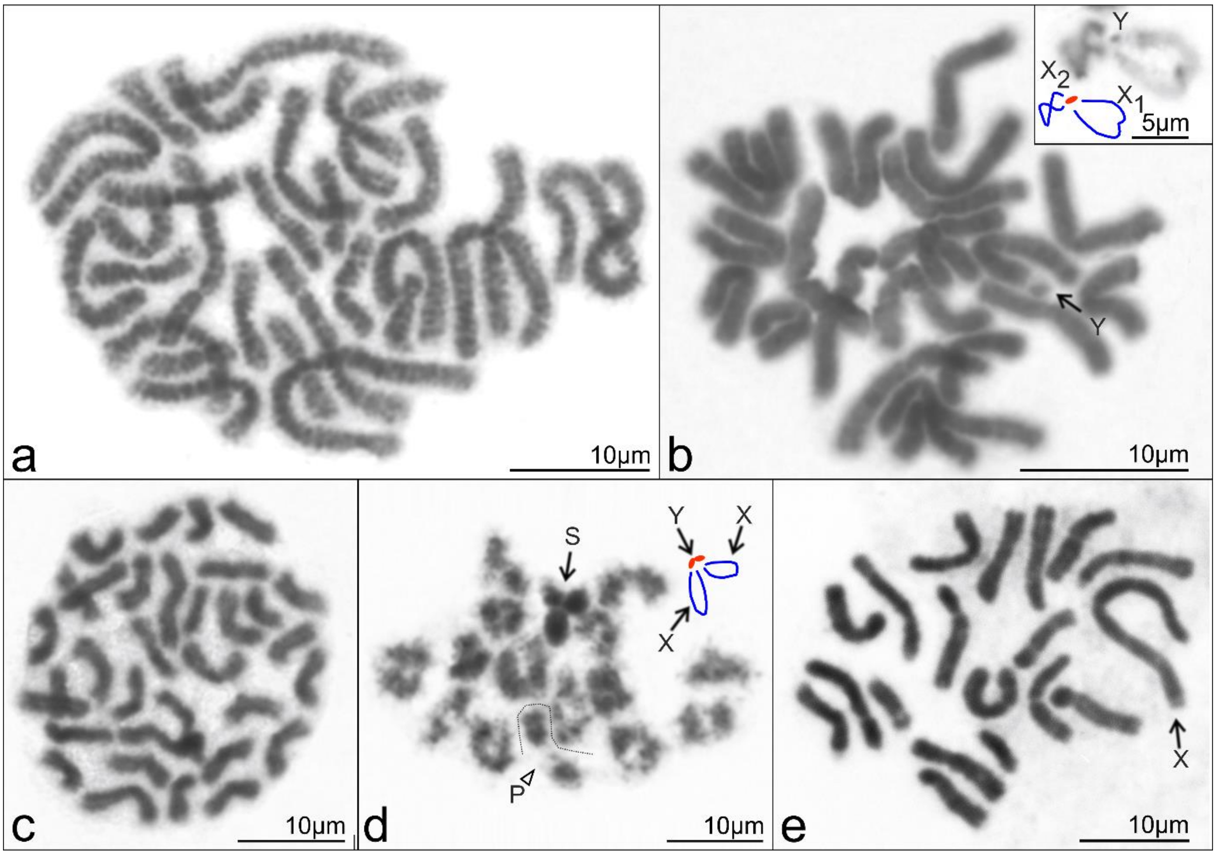

Supplementary Table 1. Collection data of specimens used.

Taxon	Locality	GPS
**Taxa with holokinetic chromosomes**		
**Dysderidae (Dysderinae)**		
Dysdera erythrina	Břežanské údolí valley, Prague-Zbraslav, Czech Republic	49°58'06.3"N 14°24'17.0"E
Dysderocrates storkani	Ivine Vodice, Velebit Mts., Croatia	44°20'11.6"N 15°32'11.8"E
Dysderocrates sp.	forest along the road from Pentalofos to Eptachori, Macedonia, Greece	40°12'42.4"N 21°03'48.3"E
Harpactocrates sp.	breeding (Spain)	-
**Dysderidae (Harpacteinae)**		
Dasumia crassitibialis	Nahal Kziv valley, Upper Galilee, Israel	33°02'29.4"N 35°10'54.4"E
Harpactea cecconii	Agios Georgios near Akamas peninsula, Cyprus	34°54'29.5"N 32°19'59.5"E
H. hentschi	gorge along the road from Olympiada to Kryovrysi, Olympos Mts., Thessaly, Greece	39°59'32.6"N 22°17'11.7"E
H. hombergi	Břežanské údolí valley, Prague-Zbraslav, Czech Republic	49°58'06.3"N 14°24'17.0"E
H. lepida	Kamenné, Trojačka Mt., Beskydy Mts., Czech Republic	49°30'20.5"N 18°00'20.2"E
H. rubicunda	Břežanské údolí valley, Prague-Zbraslav, Czech Republic	49°58'06.3"N 14°24'17.0"E
**Dysderidae (Rhodinae)**		
Kaemis sp.	Camping Can Cervera near Montseny town, Montseny Mts., Spain	41°46'12.5"N 2°24'15.1"E
**Oonopidae**		
Gamasomorpha lutzi	Luquillo, Puerto Rico, USA	18°19'23.0"N 65°44'18.7"W
Ischnothyreus sp.	Luquillo, Puerto Rico, USA	18°19'23.0"N 65°44'18.7"W
Oonops ebenecus	Luquillo, Puerto Rico, USA	18°19'23.0"N 65°44'18.7"W
O. pulcher	Antwerpen, Belgium	51°11'59.9"N 4°28'05.1"E
	Turnhout, Belgium	51°19'02.6"N 4°56'54.6"E
**Orsolobidae**		
Afrilobus sp.	*Eucalyptus*, 15 km ENE of Louwsberg on R69 road, KwaZulu-Natal, Republic of South Africa	31°08'01.0"S 25°38'06.0"E
Azanialobus sp.	Hogsback, Eastern Cape, Republic of South Africa	32°36'12.9"S 26°56'20.3"E
**Segestriidae**		
Ariadna sp.	near Okahandja (on B1 road), Namibia	22°05'38.6"S 16°57'10.0"E

Segestria bavarica	cliffs, Šárka valley, Prague, Czech Republic	50°05'43.2"N 14°19'15.1"E
S. senoculata	road from Hodslavice to Valašské Meziříčí (forest between the road and Hostašovice railway station), Czech Republic	49°31'14.8"N 18°00'51.0"E
**Taxa with monokinetic chromosomes**		
**Caponiidae (Caponiinae)**		
Caponia capensis	Benfontein Nature Reserve, Free State, Republic of South Africa	28°49'11.6"S 24°50'10.6"E
	De Hoop Nature Reserve, Western Cape, Republic of South Africa	34°27'12.2"S 20°24'15.7"E
C. hastifera	Erfenis Dam Nature Reserve, Free State, Republic of South Africa	28°30'27.8"S 26°48'24.3"E
	Hopefield farm, Bloemfontein district, Free State, Republic of South Africa	28°51'47.5"S 26°09'45.0"E
C. natalensis	Ndumo Game Reserve, KwaZulu-Natal, Republic of South Africa	26°54'23.9"S 32°19'11.1"E
**Caponiidae (Nopinae)**		
Nops aff. *variabilis*	Neiva, Colombia	2°55'05.8"N 75°17'45.0"W
Nops sp.	coffee-banana plantation with montane rainforest remains, Villa Las Neblinas, Constanza, Cordillera Central, La Vega Province, Dominican Republic	19°00'22.7"N 70°32'18.6"W
Nopsides ceralbonus	San Dionisio canyon, Sierra de la Laguna Mts., Baja California Sur State, Mexico	23°32'56.5"N 109°49'49.7"W
Tarsonops sp.	along river, La Purísima, Baja California Sur State, Mexico	26°11'16.7"N 112°06'23.4"W
**Diguetidae**		
Diguetia albolineata	breeding stock, Spiderpharm Ltd., Yarnell, AZ, USA	
**Filistatidae (Filistatinae)**		
Filistata insidiatrix	acropolis of Rhodes town, Rhodes, Greece	36°26'23.0"N 28°12'41.0"E
Kukulcania aff. *hibernalis*	La Purísima, Baja California Sur State, Mexico	26°11'16.7"N 112°06'23.4"W
Sahastata nigra	Jahel, Arava valley, Israel	30°04'53.3"N 35°07'55.6"E
**Filistatidae (Prithinae)**		
Andoharano ansieae	Sachsenheim Guest Farm, westward from Etosha, Namibia	18°44'53.5"S 17°15'22.9"E
**Pacullidae**		
Paculla sp.	Kuala Belalong Field Studies Centre, Temburong National Park, Brunei	4°32'38.9"N 115°09'40.5"E
**Pholcidae**		
Pholcus phalangioides	Prague, Czech Republic	50°04'17.8"N 14°25'26.2"E

**Scytodidae**		
Scytodes sp. 1	Adullam Nature Reserve, Nehusha, Israel	31°37'49.6"N 34°57'03.0"E
Scytodes sp. 2	Essaouira, Morocco	31°31'42.1"N 9°44'28.9"W
**Sicariidae**		
Hexophthalma sp.	approx. 20 km NE from Hentiesbaai (on B2 road), Namibia	21°56'14.5"S 14°24'28.7"E
Loxosceles rufescens	Midreshet Ben-Gurion, Israel	30°50'59.3"N 34°47'11.7"E

Supplementary Table 2. Caponiidae, karyotype data of *Caponia capensis* and *Tarsonops* sp. Abbreviations: a - acrocentric chromosomes (light blue background), CI - centromeric index, m - metacentric chromosomes (brown background), morphology - chromosome morphology, n - number of plates evaluated, pair - number of pair, RCL - relative chromosome length, SD - standard deviation, sm - submetacentric chromosomes (reddish background), st - subtelocentric chromosomes (dark blue background).

***Caponia capensis* (n = 2)**								***Tarsonops* sp. (n = 1)**			
**Pair**	**CI±SD**	**RCL±SD**	**Morphology**	**Pair**	**CI±SD**	**RCL±SD**	**Morphology**	**Pair**	**CI**	**RCL**	**Morphology**
**1**	1.27±0.45	3.81±0.20	m	**40**	9.76±0.36	1.08±0.25	a	**1**	1.10	3.31	m
**2**	1.34±0.32	3.49±0.53	m	**41**	3.68±0.34	1.05±0.38	st	**2**	1.23	3.06	m
**3**	1.14±0.28	3.21±0.36	m	**42**	9.68±0.25	1.01±0.26	a	**3**	1.39	2.91	m
**4**	2.36±0.19	3.04±0.25	sm	**43**	8.71±0.42	1.02±0.33	a	**4**	1.27	2.90	m
**5**	1.41±0.37	2.86±0.29	m	**44**	8.10±0.31	1.02±0.22	a	**5**	1.32	2.83	m
**6**	3.51±0.24	2.84±0.38	st	**45**	1.43±0.19	0.96±0.30	m	**6**	2.13	2.42	sm
**7**	2.04±0.54	2.77±0.53	sm	**46**	2.65±0.28	0.98±0.19	sm	**7**	3.49	2.30	st
**8**	1.37±0.40	2.76±0.27	m	**47**	3.47±0.46	0,94±0.57	st	**8**	1.07	2.27	m
**9**	1.62±0.33	2.73±0.45	m	**48**	8.60±0.35	0.93±0.41	a	**9**	1.37	2.22	m
**10**	1.35±0.10	2.56±0.63	m	**49**	8.19±0.16	0.93±0.52	a	**10**	1.25	1.96	m
**11**	3.53±0.63	2.37±0.47	st	**50**	9.06±0.44	0.91±0.27	a	**11**	1.09	1.70	m
**12**	1.85±0.22	2.35±0.34	sm	**51**	3.57±0.20	0.89±0.12	st	**12**	1.15	1.66	m
**13**	1.41±0.41	2.21±0.41	m	**52**	9.89±0.52	0.86±0.44	a	**13**	1.27	1.59	m
**14**	3.22±0.63	2.20±0.28	st	**53**	1.35±0.36	0.82±0.64	m	**14**	2.18	1.51	sm
**15**	3.32±0.67	2.04±0.35	st	**54**	9.10±0.12	0.82±0.37	a	**15**	2.70	1.47	sm
**16**	3.30±0.54	2.00±0.18	st	**55**	1.25±0.27	0.78±0.28	m	**16**	2.90	1.46	sm
**17**	2.59±0.38	1.99±0.22	sm	**56**	8.78±0.42	0.78±0.44	a	**17**	3.15	1.40	st
**18**	1.97±0.21	1.89±0.32	sm	**57**	9.60±0.49	0.76±0.36	a	**18**	2.11	1.36	sm
**19**	4.59±0.37	1.83±0.27	st	**58**	8.18±0.33	0.75±0.27	a	**19**	1.46	1.31	m
**20**	2.41±0.24	1.75±0.34	sm	**59**	8.36±0.26	0.73±0.35	a	**20**	1.13	1.27	m
**21**	2.34±0.19	1.64±0.53	sm	**60**	2.64±0.45	0.67±0.42	sm	**21**	1.57	1.25	m
**22**	3.49±0.30	1.60±0.39	st	**61**	9.82±0.39	0.66±0.19	a	**22**	1.26	1.19	m
**23**	2.61±0.27	1.58±0.24	sm	**62**	8.60±0.18	0.64±0.26	a	**23**	5.43	1.04	st
**24**	1.42±0.34	1.53±0.31	m	**63**	1.26±0.50	0.63±0.35	m	**24**	1.28	0.94	m
**25**	3.38±0.43	1.51±0.16	st	**64**	9.30±0.42	0.60±0.40	a	**25**	1.22	0.89	m
**26**	8.36±0.27	1.47±0.44	a	**65**	8.16±0.21	0.58±0.31	a	**26**	2.25	0.85	sm
**27**	4.19±0.11	1.47±0.37	st	**66**	8.87±0.36	0.47±0.28	a	**27**	2.54	0.84	sm
**28**	2.33±0.39	1.46±0.22	sm	**67**	9.63±0.48	0.43±0.39	a	**28**	4.95	0.81	st
**29**	9.10±0.21	1.40±0.30	a	**68**	1.12±0.19	0.37±0.22	m	**29**	2.54	0.66	sm
**30**	3.40±0.44	1.36±0.41	st					**30**	3.25	0.64	st
**31**	2.10±0.40	1.29±0.26	sm								
**32**	8.57±0.35	1.27±0.29	a								
**33**	8.63±0.52	1.25±0.44	a								
**34**	9.32±0-37	1.23±0.58	a								
**35**	3.64±0.54	1.21±0.15	st								
**36**	3.78±0.28	1.19±0.22	st								
**37**	3.55±0.33	1.15±0.42	st								
**38**	8.78±0.47	1.15±0.39	a								
**39**	8.63±0.39	1.09±0.17	a								

Supplementary Table 3. Summary of karyotype and genome data used for the preparation of Figure 4. Abbreviations: structure - chromosome structure (H - holokinetic; M - monocentric), 2C - DNA content of diploid chromosome complements in Mbp, 2C/2n - average chromosome size (i.e., genome size/chromosome number, Mbp/chromatid), GC - GC proportion (%).

Species	Morphology	Clade	Clade number	2n	2C	GC	2C/2n
Filistata insidiatrix	M	Filistatidae	1	34♀	8521.09	36.342	250.62
Kukulcania aff. *hibernalis*	M	Filistatidae	1	26♀	10259.4	32.257	394.591
Sahastata nigra	M	Filistatidae	1	28♀	11849.2	33.679	423.186
Andorahano ansieae	M	Filistatidae	1	24♀	5111.78	35.899	212.991
Hexopthalma sp.	M	Scytodidae + Sicariidae	2	20♀	2587.14	36.096	129.357
Loxosceles rufescens	M	Scytodidae + Sicariidae	2	22♀	10182.8	39.807	462.854
Scytodes sp. 1	M	Scytodidae + Sicariidae	2	20♀	4551.01	42.579	227.55
Scytodes sp. 2	M	Scytodidae + Sicariidae	2		3057.96	39.826	
Diguetia albolineata	M	Diguetidae + Pacullidae + Pholcidae	3	20♀	2954.35	43.542	147.717
Paculla sp.	M	Diguetidae + Pacullidae + Pholcidae	3	34♀	7658.65	40.583	225.255
Pholcus phalangioides	M	Diguetidae + Pacullidae + Pholcidae	3	26♀	1754.41	34.555	67.4774
Caponia natalensis	M	Caponiidae	4	158♀			
Caponia capensis	M	Caponiidae	4	136♀	38927.5		286.232
Caponia hastifera	M	Caponiidae	4	136♀	47428.6	43.183	348.739
Nops aff. *variabilis*	M	Caponiidae	4	55♂	31121.4	42.246	565.844
Nops sp.	M	Caponiidae	4	62♀	32830	43.525	529.517
Nopsides ceralbonus	M	Caponiidae	4	64♀			
Tarsonops sp.	M	Caponiidae	4	60♀			
Ariadna sp.	H	Segestriidae	5	8♀	16890.7	39.351	2111.33
Segestria bavarica	H	Segestriidae	5	16♀	6251.37	38.488	390.711
Segestria senoculata	H	Segestriidae	5	16♀	8043.74	38.94	502.734
Gamasomorpha lutzi	H	Oonopidae	6	8♀			
Ischnothyreus sp.	H	Oonopidae	6	8♀			
Oonops ebenecus	H	Oonopidae	6	8♀			
Oonops pulcher	H	Oonopidae	6	8♀	6920.37	36.693	865.046
Afrilobus sp.	H	Orsolobidae	7	6♀			

Species	Morphology	Clade	Clade number	2n	2C	GC	2C/2n
Azanialobus sp.	H	Orsolobidae	7		3581.92		
Dasumia crassitibialis	H	Harpacteinae	8	8♀	5824.28	37.088	728.036
Harpactea cecconii	H	Harpacteinae	8	8♀			
Harpactea hentschi	H	Harpacteinae	8	8♀	9567	39.335	1195.87
Harpactea hombergi	H	Harpacteinae	8	8♀			
Harpactea lepida	H	Harpacteinae	8	26♀	8223.23	39.106	316.278
Harpactea rubicunda	H	Harpacteinae	8	8♀	6243.5	39.369	780.438
Dysdera erythrina	H	Dysderinae	9	20♀	7644.37	39.25	382.218
Dysderocrates storkani	H	Dysderinae	9	22♀			
Dysderocrates sp.	H	Dysderinae	9	10♀	3137.16	36.458	313.716
Harpactocrates sp.	H	Dysderinae	9	10♀			
